# New Diagnosis Test under the Neutrosophic Statistics: An Application to Diabetic Patients

**DOI:** 10.1155/2020/2086185

**Published:** 2020-01-22

**Authors:** Muhammad Aslam, Osama H. Arif, Rehan Ahmad Khan Sherwani

**Affiliations:** ^1^Department of Statistics, Faculty of Science, King Abdulaziz University, Jeddah 21551, Saudi Arabia; ^2^College of Statistical and Actuarial Sciences, University of the Punjab, Lahore, Pakistan

## Abstract

The diagnosis tests (DT) under classical statistics are applied under the assumption that all observations in the data are determined. Therefore, these DT cannot be applied for the analysis of the data when some or all observations are not determined. The neutrosophic statistics (NS) which is the extension of classical statistics can be applied for the data having uncertain, unclear, and fuzzy observations. In this paper, we will present the DT, and gold-standard tests under NS are called neutrosophic diagnosis tests (NDT). Therefore, the proposed NDT is the generalization of the existing DT and can be applied under the uncertainty environment. We will present the NDT table and present a real example from the medical field. The use of the proposed method will be more effective and adequate to be used in medical science, biostatistics, decision, and classification analysis.

## 1. Introduction

Classical statistics (CS) has been widely applied for the presentations, analysis and inference of the data in a variety of fields. The CS makes the analysis under the assumption that the observations recorded in the data should be determined. Among many statistical tests, the diagnosis tests (DT) have been widely used in medical science and biostatistics for the analysis of the data. The test provides the measures of sensitivity and specificity of the test using the data presented in the contingency table. Several authors used these diagnostic tests in the variety of fields. Greiner et al. [1] applied these tests for the analysis of the veterinary data. Lalkhen et al. [2] discussed these tests for clinical data. Parikh et al. [3] discussed the application of DT in medical science. van Stralen et al. [4] applied DT on the kidney data. Leeflang et al. [5] used DT to analyze the disease data.

The fuzzy logic is applied when the data analyst is not sure about some observations or parameters. The DT under the fuzzy approach has been widely used to analyze the data having uncertain values. Phelps and Hutson [6] worked on DT under fuzzy logic. Castanho et al. [7] studied the operating characteristics curve for the DT. Hashmia and kanb [8] used these tests for liver disease data. Smith and Slenning [9] and Bhise et al. [10] provided the DT for the uncertainty environment. More applications of the DTs can be seen in [8].

The neutrosophic logic is introduced by Smarandache [11] and considered as the extension of the fuzzy logic. The neutrosophic logic considered the measure of indeterminacy addition to the measures of truthiness and falseness. For more details on neutrosophic logic, the reader may refer [12–14]. In practice, when the data are obtained using some tools, it may be possible to present some observations in a range. Therefore, CS cannot be used for the analysis of the data given in the indeterminacy interval. The neutrosophic statistics (NS) is the alternative of CS to be applied under the uncertainty environment. The NS is based on neutrosophic numbers. The NS logic is the extension of the fuzzy logic and deals with the measure of indeterminacy, see [15]. Therefore, the NS is the extension of CS and can be applied for the analysis of the data when data are selected from the population having uncertain, fuzzy, and imprecise observations.

The DT given in the literature cannot be applied under the uncertainty environment. In this paper, we will present the DT and gold-standard tests under NS, called neutrosophic diagnosis tests (NDT). Therefore, the proposed NDT is the generalization of the existing DT and can be applied under the uncertainty environment. We will present the NDT table and present a real example from the medical field. The use of the proposed method will be more effective and adequate to be used in medical science, biostatistics, and decision and for classification analysis.

## 2. Proposed Diagnosis Tests

In this section, we present a diagnosis table under the neutrosophic statistical interval method. We will present some important formulas for the DT and gold-standard tests under the NS.  Table 1 is given for the true diagnosis and test results under the NS. Note here that Table 1 under NS reduces to DT under CS when all observations in population or the sample are determined. Therefore, the proposed DTs given in Table 1 are the generalizations of DT under CS.

Based on the information given in Table 1, we have the following formulas to find the necessary measures for the diagnosis tests.

The proportion of diseased persons correctly identified by the test having that particular disease under the uncertainty environment is called Neutrosophic sensitivity and denoted by  *P*_*N*_(+ve/*D*). It is defined as Neutrosophic sensitivity:

(1) $$\begin{array}{c} N\text{ Sens}=P_{N}\left(\frac{+\text{ve}}{D}\right)=\frac{\left[a_{L},a_{U}\right]}{\left[a_{L}+e_{L}+i_{L}+m_{L},a_{U}+e_{U}+i_{U}+m_{U}\right]}. \end{array}$$

The proportion of persons that the practitioner accepts to have the disease with uncertainty and the test indicates the presence of disease is called Neutrosophic practitioner sensitivity and denoted by  *P*_*N*_(+ve/Prac+ve). It is defined as Neutrosophic sensitivity:

(2) $$\begin{array}{c} N\text{ prac Sens}=P_{N}\left(\frac{+\text{ve}}{\text{Prac}+\text{ve}}\right)=\frac{\left[c_{L},c_{U}\right]}{\left[c_{L}+g_{L}+k_{L}+o_{L},c_{U}+g_{U}+k_{U}+o_{U}\right]}. \end{array}$$

The proportion of persons having disease for which the practitioner accepts with uncertainty the test results is called Neutrosophic test sensitivity and denoted by  *P*_*N*_(Test+ve/+ve). It is defined as Neutrosophic sensitivity:

(3) $$\begin{array}{c} N\text{ Test Sens}=P_{N}\left(\frac{\text{Test}+\text{ve}}{+\text{ve}}\right)=\frac{\left[i_{L},i_{U}\right]}{\left[a_{L}+e_{L}+i_{L}+m_{L},a_{U}+e_{U}+i_{U}+m_{U}\right]}. \end{array}$$

The proportion of persons for which the practitioner and the test both are uncertain is called Neutrosophic practitioner-test sensitivity and denoted by  *P*_*N*_(Prac Test+ve/+ve). It is defined as: Neutrosophic sensitivity:

(4) $$\begin{array}{c} N\text{ Prac Test Sens}=P_{N}\left(\frac{\text{Prac Test}+\text{ve}}{+\text{ve}}\right)=\frac{\left[k_{L},k_{U}\right]}{\left[c_{L}+g_{L}+k_{L}+o_{L},c_{U}+g_{U}+k_{U}+o_{U}\right]}. \end{array}$$

The proportion of nondiseased persons correctly identified by the test not having that particular disease under the uncertainty environment is called Neutrosophic specificity and denoted by  *P*_*N*_(−ve/ND). It is defined as Neutrosophic specificity:

(5) $$\begin{array}{c} N\text{ Spec }=P_{N}\left(\frac{-\text{ve}}{\text{ND}}\right)=\frac{\left[f_{L},f_{U}\right]}{\left[b_{L}+f_{L}+j_{L}+n_{L},b_{U}+f_{U}+j_{U}+n_{u}\right]}. \end{array}$$

The proportion of persons that the practitioner accepts having no disease with uncertainty and the test indicates the nonpresence of disease is called Neutrosophic practitioner specificity and denoted by  *P*_*N*_(−ve/Prac − ve). It is defined as Neutrosophic specificity:

(6) $$\begin{array}{c} N\text{ prac Spec}=P_{N}\left(\frac{-\text{ve}}{\text{Prac}-\text{ve}}\right)=\frac{\left[h_{L},h_{U}\right]}{\left[d_{L}+h_{L}+l_{L}+p_{L},d_{U}+h_{U}+l_{U}+p_{U}\right]}. \end{array}$$

The proportion of persons having no disease but the practitioner accepts with uncertainty the test results is called Neutrosophic test specificity and denoted by  *P*_*N*_(Test − ve/−ve). It is defined as Neutrosophic specificity:

(7) $$\begin{array}{c} N\text{ Test Spec}=P_{N}\left(\frac{\text{Test}-\text{ve}}{-\text{ve}}\right)=\frac{\left[n_{L},n_{u}\right]}{\left[b_{L}+f_{L}+j_{L}+n_{L},b_{U}+f_{U}+j_{U}+n_{u}\right]}. \end{array}$$

The proportion of persons for which the practitioner and the test both are uncertain about the disease is called Neutrosophic practitioner-test specificity and denoted by  *P*_*N*_(−ve/Prac Test − ve). It is defined as Neutrosophic sensitivity:

(8) $$\begin{array}{c} N\text{ Prac Test Spec}=P_{N}\left(\frac{\text{Prac Test}-\text{ve}}{-\text{ve}}\right)=\frac{\left[p_{L},p_{U}\right]}{\left[d_{L}+h_{L}+l_{L}+p_{L},d_{U}+h_{U}+l_{U}+p_{U}\right]}. \end{array}$$

The proportion of persons with positive test results when actually the persons have the particular disease under the uncertainty environment is called Neutrosophic positive predictive value (NPPV) and denoted by  *P*_*N*_(*D*/+*ve*). It is defined as Neutrosophic positive predictive value:

(9) $$\begin{array}{c} \text{NPPV}=P_{N}\left(+ve/D\right)=\frac{\left[a_{L},a_{U}\right]}{\left[a_{L}+b_{L}+c_{L}+d_{L},a_{U}+b_{U}+c_{U}+d_{U}\right]}. \end{array}$$

The proportion of diseased persons for which the practitioner accepts the persons having a disease with uncertainty is called Neutrosophic practitioner positive predictive value and denoted by  *P*_*N*_(Prac+ve/+ve). It is defined as.

Neutrosophic practitioner positive predictive:

(10) $$\begin{array}{c} \text{NPPPV}=P_{N}\left(\frac{\text{Prac}+\text{ve}}{+\text{ve}}\right)=\frac{\left[c_{L},c_{U}\right]}{\left[a_{L}+b_{L}+c_{L}+d_{L},a_{U}+b_{U}+c_{U}+d_{U}\right]}. \end{array}$$

The proportion of persons for which the test is positive with uncertainty and actually the persons have the disease is called Neutrosophic test positive predictive value (NTPPV) and denoted by  *P*_*N*_(+ve/Test+ve). It is defined as Neutrosophic test positive predictive value:

(11) $$\begin{array}{c} \text{NTPPV}=P_{N}\left(\frac{+\text{ve}}{\text{Test}+\text{ve}}\right)=\frac{\left[i_{L},i_{U}\right]}{\left[i_{L}+j_{L}+k_{L}+l_{L},i_{U}+j_{U}+k_{U}+l_{U}\right]}. \end{array}$$

The proportion of persons for which test results and the practitioner both are uncertain is called Neutrosophic practitioner-test sensitivity and denoted by  *P*_*N*_(+ve/Prac Test+ve). It is defined as Neutrosophic sensitivity:

(12) $$\begin{array}{c} N\text{ Prac Test Sens}=P_{N}\left(\frac{+\text{ve}}{\text{Prac Test}+\text{ve}}\right)=\frac{\left[k_{L},k_{U}\right]}{\left[i_{L}+j_{L}+k_{L}+l_{L},i_{U}+j_{U}+k_{U}+l_{U}\right]}. \end{array}$$

The proportion of persons with the negative test results when actually they have no disease under the uncertainty environment is called Neutrosophic negative predictive value (NNPV) denoted by  *P*_*N*_(ND/−ve). It is defined as Neutrosophic specificity:

(13) $$\begin{array}{c} N\text{ Spec}=P_{N}\left(\frac{\text{ND}}{-\text{ve}}\right)=\frac{\left[f_{L},f_{U}\right]}{\left[e_{L}+f_{L}+g_{L}+h_{L},e_{U}+f_{U}+g_{U}+h_{U}\right]}. \end{array}$$

The proportion of persons for whom test results are negative and the practitioner accepts no disease with uncertainty is called Neutrosophic practitioner predictive negative value (NPPNV) denoted by  *P*_*N*_(Prac − ve/−ve). It is defined as Neutrosophic specificity:

(14) $$\begin{array}{c} N\text{ prac Spec}=P_{N}\left(\frac{\text{Prac}-\text{ve}}{-\text{ve}}\right)=\frac{\left[h_{L},h_{U}\right]}{\left[e_{L}+f_{L}+g_{L}+h_{L},e_{U}+f_{U}+g_{U}+h_{U}\right]}. \end{array}$$

The proportion of persons with negative test under uncertainty when actually do not having the disease is called Neutrosophic test predictive value (NTPV) and denoted by  *P*_*N*_(−ve/Test − *ve*). It is defined as Neutrosophic specificity:

(15) $$\begin{array}{c} N\text{ Test Spec}=P_{N}\left(\frac{-\text{ve}}{\text{Test}-\text{ve}}\right)=\frac{\left[n_{L},n_{u}\right]}{\left[m_{L}+n_{L}+o_{L}+p_{L},m_{U}+n_{U}+o_{U}+p_{U}\right]}. \end{array}$$

The proportion of persons with negative results and the practitioner disagrees with uncertainty is called Neutrosophic practitioner-test specificity and denoted by  *P*_*N*_(−ve/Prac Test − ve). It is defined as Neutrosophic sensitivity:

(16) $$\begin{array}{c} N\text{ Prac Test Spec}=P_{N}\left(\frac{\text{Prac Test}-\text{ve}}{-\text{ve}}\right)=\frac{\left[p_{L},p_{U}\right]}{\left[m_{L}+n_{L}+o_{L}+p_{L},m_{U}+n_{U}+o_{U}+p_{U}\right]}. \end{array}$$

The neutrosophic-positive likelihood ratio:

(17) $$\begin{array}{c} {\text{NLR}}^{+}=\frac{N\text{ Sens}}{1-N\text{ Spec}}. \end{array}$$

The neutrosophic-positive practitioner likelihood ratio:

(18) $$\begin{array}{c} {\text{NPLR}}^{+}=\frac{N\text{ prac Sens}}{1-N\text{ prac Spec}}. \end{array}$$

The neutrosophic-positive test likelihood ratio:

(19) $$\begin{array}{c} {\text{NTLR}}^{+}=\frac{N\text{ Test Sens}}{1-N\text{ Test Spec}}. \end{array}$$

The neutrosophic-positive practitioner-test likelihood ratio:

(20) $$\begin{array}{c} {\text{NPTLR}}^{+}=\frac{N\text{ Prac Test Sens}}{1-N\text{ Prac Test Specc}}. \end{array}$$

The neutrosophic-negative likelihood ratio:

(21) $$\begin{array}{c} {\text{NLR}}^{-}=\frac{1-N\text{ Spec}}{N\text{ Sens}}. \end{array}$$

The neutrosophic-negative practitioner likelihood ratio:

(22) $$\begin{array}{c} {\text{NPLR}}^{-}=\frac{1-N\text{ prac Spec}}{N\text{ prac Sens}}. \end{array}$$

The neutrosophic-negative test likelihood ratio:

(23) $$\begin{array}{c} {\text{NTLR}}^{-}=\frac{1-N\text{ Test Spec}}{N\text{ Test Sens}}. \end{array}$$

The neutrosophic-negative practitioner-test likelihood ratio:

(24) $$\begin{array}{c} {\text{NPTLR}}^{-}=\frac{1-N\text{ Prac Test Specc}}{N\text{ Prac Test Sens}}. \end{array}$$

## 3. Example

In this section, we discuss the application of the proposed DT under the NS with the help of a data taken from medical science. The purpose of this study is to classify whether the patient under the investigation has the disease or not. The practitioner is interested to detect the disease early. We consider a test for diabetes to assess the status of the sugar level in the patients. If the test has a positive result, then the patient presumed the diabetic patient. Note here that sometimes, the diagnosis test does not clearly indicate the presence of the disease or the practitioner is not sure about the test results or about the true diagnosis. In Table 1, we also introduced these categories under the uncertainty environment. We will perform the gold-standard tests to see either the patient has the disease or not. In Table 2, we present the simulated data of diabetes patients under the NS.

The important measures for the data presented in Table 2 are given below.

Neutrosophic sensitivity:

(25) $$\begin{array}{c} N\text{ Sens}=P_{N}\left(+\text{ve}/D\right)=\frac{\left[a_{L},a_{U}\right]}{\left[a_{L}+e_{L}+i_{L}+m_{L},a_{U}+e_{U}+i_{U}+m_{U}\right]}=\;\left[0.6651,0.6637\right]\;=\left[66.51\%,66.37\%\right]. \end{array}$$

Neutrosophic sensitivity:

(26) $$\begin{array}{c} N\text{ prac Sens}=P_{N}\left(+\text{ve}/\text{Prac}+\text{ve}\right)=\frac{\left[c_{L},c_{U}\right]}{\left[c_{L}+g_{L}+k_{L}+o_{L},c_{U}+g_{U}+k_{U}+o_{U}\right]}=\left[0.1904,0.0975\right]=\left[19.04\%,9.75\%\right]. \end{array}$$

Neutrosophic sensitivity:

(27) $$\begin{array}{c} N\text{ Test Sens}=P_{N}\left(\text{Test}+\text{ve}/+\text{ve}\right)=\frac{\left[i_{L},i_{U}\right]}{\left[a_{L}+e_{L}+i_{L}+m_{L},a_{U}+e_{U}+i_{U}+m_{U}\right]}=\left[0.000739,0.001472\right]=\left[0.0739\%,0.1471\%\right]. \end{array}$$

Neutrosophic sensitivity:

(28) $$\begin{array}{c} N\text{ Prac Test Sens}=P_{N}\left(\text{Prac Test}+\text{ve}/+\text{ve}\right)=\frac{\left[k_{L},k_{U}\right]}{\left[c_{L}+g_{L}+k_{L}+o_{L},c_{U}+g_{U}+k_{U}+o_{U}\right]}=\left[0.4761,0.2682\right]=\left[47.61\%,26.82\%\right]. \end{array}$$

Neutrosophic specificity:

(29) $$\begin{array}{c} N\text{ Spec}=P_{N}\left(-\text{ve}/\text{ND}\right)=\frac{\left[f_{L},f_{U}\right]}{\left[b_{L}+f_{L}+j_{L}+n_{L},b_{U}+f_{U}+j_{U}+n_{u}\right]}=\left[0.7619,0.7614\right]=\left[76.19\%,76.14\%\right]. \end{array}$$

Neutrosophic specificity:

(30) $$\begin{array}{c} N\text{ prac Spec}=P_{N}\left(-\text{ve}/\text{Prac}-\text{ve}\right)=\frac{\left[h_{L},h_{U}\right]}{\left[d_{L}+h_{L}+l_{L}+p_{L},d_{U}+h_{U}+l_{U}+p_{U}\right]}=\left[0.1935,0.2121\right]=\left[19.35\%,21.21\%\right]. \end{array}$$

Neutrosophic specificity:

(31) $$\begin{array}{c} N\text{ Test Spec}=P_{N}\left(\text{Test}-\text{ve}/-\text{ve}\right)=\frac{\left[n_{L},n_{u}\right]}{\left[b_{L}+f_{L}+j_{L}+n_{L},b_{U}+f_{U}+j_{U}+n_{u}\right]}=\left[0.0.0010,0.0019\right]=\left[0.1073\%,0.1927\%\right]. \end{array}$$

Neutrosophic sensitivity:

(32) $$\begin{array}{c} N\text{ Prac Test Spec}=P_{N}\left(\text{Prac Test}-\text{ve}/-\text{ve}\right)=\frac{\left[p_{L},p_{U}\right]}{\left[d_{L}+h_{L}+l_{L}+p_{L},d_{U}+h_{U}+l_{U}+p_{U}\right]}=\left[0.1612,0.1818\right]=\left[16.12\%,18.18\%\right]. \end{array}$$

Neutrosophic positive predictive value:

(33) $$\begin{array}{c} \text{NPPV}=P_{N}\left(+\text{ve}/D\right)=\frac{\left[a_{L},a_{U}\right]}{\left[a_{L}+b_{L}+c_{L}+d_{L},a_{U}+b_{U}+c_{U}+d_{U}\right]}=\left[0.4479,0.4485\right]=\left[44.79\%,44.85\%\right]. \end{array}$$

Neutrosophic practitioner-positive predictive:

(34) $$\begin{array}{c} \text{NPPPV}=P_{N}\left(\text{Prac}+\text{ve}/+\text{ve}\right)=\frac{\left[c_{L},c_{U}\right]}{\left[a_{L}+b_{L}+c_{L}+d_{L},a_{U}+b_{U}+c_{U}+d_{U}\right]}=\left[0.0024,0.0024\right]=\left[0.24\%,0.24\%\right]. \end{array}$$

Neutrosophic test-positive predictive value:

(35) $$\begin{array}{c} \text{NTPPV}=P_{N}\left(+\text{ve}/\text{Test}+\text{ve}\right)=\frac{\left[i_{L},i_{U}\right]}{\left[i_{L}+j_{L}+k_{L}+l_{L},i_{U}+j_{U}+k_{U}+l_{U}\right]}=\left[0.0333,0.0606\right]=\left[3.3333\%,6.0606\%\right]. \end{array}$$

Neutrosophic sensitivity:

(36) $$\begin{array}{c} N\text{ Prac Test Sens}=P_{N}\left(+\text{ve}/\text{Prac Test}+\text{ve}\right)=\frac{\left[k_{L},k_{U}\right]}{\left[i_{L}+j_{L}+k_{L}+l_{L},i_{U}+j_{U}+k_{U}+l_{U}\right]}=\left[0.3333,0.3333\right]=\left[33.33\%,33.33\%\right]. \end{array}$$

Neutrosophic specificity:

(37) $$\begin{array}{c} N\text{ Spec}=P_{N}\left(\text{ND}/-\text{ve}\right)=\frac{\left[f_{L},f_{U}\right]}{\left[e_{L}+f_{L}+g_{L}+h_{L},e_{U}+f_{U}+g_{U}+h_{U}\right]}=\;\left[0.8857,0.8854\right]. \end{array}$$

Neutrosophic specificity:

(38) $$\begin{array}{c} N\text{ prac Spec}=P_{N}\left(\text{Prac}-\text{ve}/-\text{ve}\right)=\frac{\left[h_{L},h_{U}\right]}{\left[e_{L}+f_{L}+g_{L}+h_{L},e_{U}+f_{U}+g_{U}+h_{U}\right]}=\left[0.1497,0.0155\right]=\left[0.1497\%,1.55\%\right]. \end{array}$$

Neutrosophic specificity:

(39) $$\begin{array}{c} N\text{ Test Spec}=P_{N}\left(-\text{ve}/\text{Test}-\text{ve}\right)=\frac{\left[n_{L},n_{u}\right]}{\left[m_{L}+n_{L}+o_{L}+p_{L},m_{U}+n_{U}+o_{U}+p_{U}\right]}=\left[0.1515,0.2093\right]=\left[15.15\%,20.93\%\right]. \end{array}$$

Neutrosophic sensitivity:

(40) $$\begin{array}{c} N\text{ Prac Test Spec}=P_{N}\left(\text{Prac Test}-\text{ve}/-\text{ve}\right)=\frac{\left[p_{L},p_{U}\right]}{\left[m_{L}+n_{L}+o_{L}+p_{L},m_{U}+n_{U}+o_{U}+p_{U}\right]}=\left[0.1515,0.1395\right]=\left[15.15\%,13.95\%\right]. \end{array}$$

The neutrosophic-positive likelihood ratio:

(41) $$\begin{array}{c} {\text{NLR}}^{+}=\frac{N\text{ Sens}}{1-N\text{ Spec}}=\frac{\left[0.6651,0.6637\right]}{1-\left[0.7619,0.7614\right]}=\;\left[2.79,2.78\right]. \end{array}$$

The neutrosophic-positive practitioner likelihood ratio:

(42) $$\begin{array}{c} {\text{NPLR}}^{+}=\frac{N\text{ prac Sens}}{1-N\text{ prac Spec}}=\frac{\left[0.1904,0.0975\right]}{1-\left[0.1935,0.2121\right]}=\;\left[0.2360,0.1237\right]. \end{array}$$

The neutrosophic-positive test likelihood ratio:

(43) $$\begin{array}{c} {\text{NTLR}}^{+}=\frac{N\text{ Test Sens}}{1-N\text{ Test Spec}}=\frac{\left[0.000739,\;0.001472\right]}{1-\;\left[0.0.0010,0.0019\right]}=\left[0.0007,0.0014\right]. \end{array}$$

The neutrosophic-positive practitioner-test likelihood ratio:

(44) $$\begin{array}{c} {\text{NPTLR}}^{+}=\frac{N\text{ Prac Test Sens}}{1-N\text{ Prac Test Specc}}=\frac{\left[0.4761,0.2682\right]}{1-\left[0.1612,0.1818\right]}=\;\left[0.5675,0.3277\right]. \end{array}$$

The neutrosophic-negative likelihood ratio:

(45) $$\begin{array}{c} {\text{NLR}}^{-}=\frac{1-N\text{ Spec}}{N\text{ Sens}}=\frac{1-\left[0.7619,0.7614\right]}{\left[0.6651,0.6637\right]}=\;\left[0.3579,0.3595\right]. \end{array}$$

The neutrosophic-negative practitioner likelihood ratio:

(46) $$\begin{array}{c} {\text{NPLR}}^{-}=\frac{1-N\text{ prac Spec}}{N\text{ prac Sens}}=\frac{1-\left[0.1935,0.2121\right]}{\left[0.1904,0.0975\right]}=\left[4.23,8.08\right]. \end{array}$$

The neutrosophic-negative test likelihood ratio:

(47) $$\begin{array}{c} {\text{NTLR}}^{-}=\frac{1-\;\left[0.0.0010,0.0019\right]}{\left[0.000739,\;0.001472\right]}=\left[1351.82,678.05\right]. \end{array}$$

The neutrosophic-negative practitioner-test likelihood ratio:

(48) $$\begin{array}{c} {\text{NPTLR}}^{-}=\frac{1-\left[0.1612,0.1818\right]}{\left[0.4761,0.2682\right]}=\left[1.76,3.05\right]. \end{array}$$

## 4. Discussion of Results

Based on the calculations for the data given in Table 2, we note that the indeterminacy interval in neutrosophic sensitivity and septicity are 66.51% and 66.37% and 76.19% and 76.14%, respectively. This means, the patient truly has the disease and the test will return a positive result from 66.37% to 66.51% the times. Similarly, the patient has no disease and the test will return a negative result from 76.14% to 76.19% the times. The *N* prac Sens is from 9.75% to 19.04%. It means that the practitioner certainty about the disease is from 9.75% to 19.04% when the test will return the positive result. The proportion of persons having the disease and the practitioner accepts with uncertainty the test is from 0.0739% to 0.1471%. The person having positive or negative results and the chance of having diabetes or not is from NPPV*ɛ* [44.79%,  44.85%]. Similarly, the other measure can be interpreted. The values of NLR^+^*ɛ*[2.79,  2.78] > 1 indicates that the test does indicate the positive results correctly. The indeterminacy interval of NPLR^+^*ɛ*[0.2360,  0.1237] < 1 indicates that practitioner experience is good in diagnosis of the disease. Similarly, other measures can be interpreted.

## 5. Concluding Remarks

In this paper, we presented the DT and gold-standard tests under NS, called neutrosophic diagnosis tests (NDT). Therefore, the proposed NDT was the generalization of the existing DT and can be applied under the uncertainty environment. We presented the NDT table and present a real example from the medical field. From the real example, it is concluded that the proposed NDT are considered results when the practitioner is uncertain about the true diagnosis or the test results. The proposed tests are recommended to use for the analysis under uncertainty. The use of the proposed method will be more effective and adequate to be used in medical science, biostatistics, and decision and for classification analysis.

## Figures and Tables

**Table 1:** The DT under the NS.

	True diagnosis					
Test results		Disease +ve	Disease −ve	Uncertainty +ve	Uncertainty −ve	Total
	+ve	[*a*_*L*_, *a*_*U*_]	[*b*_*L*_, *b*_*U*_]	[*c*_*L*_, *c*_*U*_]	[*d*_*L*_, *d*_*U*_]	[*a*_*L*_+*b*_*L*_+*c*_*L*_+*d*_*L*_, *a*_*U*_+*b*_*U*_+*c*_*U*_+*d*_*U*_]
	−ve	[*e*_*L*_, *e*_*U*_]	[*f*_*L*_, *f*_*U*_]	[*g*_*L*_, *g*_*U*_]	[*h*_*L*_, *h*_*U*_]	[*e*_*L*_+*f*_*L*_+*g*_*L*_+*h*_*L*_, *e*_*U*_+*f*_*U*_+*g*_*U*_+*h*_*U*_]
	Uncertainty +ve	[*i*_*L*_, *i*_*U*_]	[*j*_*L*_, *j*_*U*_]	[*k*_*L*_, *k*_*U*_]	[*l*_*L*_, *l*_*U*_]	[*i*_*L*_+*j*_*L*_+*k*_*L*_+*l*_*L*_, *i*_*U*_+*j*_*U*_+*k*_*U*_+*l*_*U*_]
	Uncertainty −ve	[*m*_*L*_, *m*_*U*_]	[*n*_*L*_, *n*_*u*_]	[*o*_*L*_, *o*_*U*_]	[*p*_*L*_, *p*_*U*_]	[*m*_*L*_+*n*_*L*_+*o*_*L*_+*p*_*L*_, *m*_*U*_+*n*_*U*_+*o*_*U*_+*p*_*U*_]
Totals		[*a*_*L*_+*e*_*L*_+*i*_*L*_+*m*_*L*_, *a*_*U*_+*e*_*U*_+*i*_*U*_+*m*_*U*_]	[*b*_*L*_+*f*_*L*_+*j*_*L*_+*n*_*L*_, *b*_*U*_+*f*_*U*_+*j*_*U*_+*n*_*u*_]	[*c*_*L*_+*g*_*L*_+*k*_*L*_+*o*_*L*_, *c*_*U*_+*g*_*U*_+*k*_*U*_+*o*_*U*_]	[*d*_*L*_+*h*_*L*_+*l*_*L*_+*p*_*L*_, *d*_*U*_+*h*_*U*_+*l*_*U*_+*p*_*U*_]	*N*_*N*_

**Table 2:** The real data under NS.

	True diagnosis					
Test results		Diabetic	Not diabetic	Uncertainty +ve	Uncertainty −ve	Total
	+ve	[900, 902]	[1100, 1100]	[4, 4]	[5, 5]	[2009, 2011]
	−ve	[450, 450]	[3550, 3555]	[2, 3]	[6, 7]	[4008, 4015]
	Uncertainty + ve	[1, 2]	[4, 5]	[10, 11]	[15, 15]	[30, 33]
	Uncertainty −ve	[2, 5]	[5, 9]	[21, 23]	[5, 6]	[33, 43]
Totals	[1353, 1359]	[4659, 4669]	[37, 41]	[31, 33]	[6080, 6102]	

## Data Availability

The data are given in the paper.
